# Correlation of progression-free survival and overall survival after treatment for relapsed Hodgkin lymphoma: individual patient data analysis of randomized German Hodgkin Study Group (GHSG) Trials

**DOI:** 10.1038/s41375-025-02567-w

**Published:** 2025-03-24

**Authors:** P. J. Bröckelmann, H. Müller, M. Fuchs, S. Gillessen, D. A. Eichenauer, S. Borchmann, A. S. Robertz, K. Behringer, J. Welters, J. Ferdinandus, B. Böll, H. Tharmaseelan, X. Yang, C. Kobe, H. -T. Eich, C. Baues, W. Klapper, P. Borchmann, B. von Tresckow

**Affiliations:** 1 University of Cologne, Faculty of Medicine and University Hospital of Cologne, Department I of Internal Medicine, Center for Integrated Oncology Aachen Bonn Cologne Düsseldorf (CIO ABCD) and German Hodgkin Study Group (GHSG), Cologne, Germany; 2 Merck & Co., Inc, Kenilworth, NJ USA; 3 Department of Nuclear Medicine, University Hospital of Cologne, Cologne, Germany; 4 Department of Radiation Oncology, University Hospital of Muenster, Muenster, Germany; 5 Department of Radiotherapy, University of Bochum, Bochum, Germany; 6 Hematopathology Section and Lymph Node Registry, Department of Pathology, University Hospital Schleswig-Holstein, Campus Kiel, Kiel, Germany; 7 Department of Hematology and Stem Cell Transplantation, West German Cancer Center and German Cancer Consortium (DKTK partner site Essen), University Hospital Essen, University of Duisburg-Essen, Essen, Germany

**Keywords:** Hodgkin lymphoma, Clinical trials

## To the Editor:

Progression-free survival (PFS) and overall survival (OS) are key indicators of treatment success in Hodgkin lymphoma (HL), both in the context of first-line treatment and in relapsed HL. While OS is often prioritized by regulatory authorities and health technology assessment bodies [1], PFS is highly relevant to patients and frequently serves as primary endpoint in clinical research [2]. For a given patient, avoiding a PFS event not only means being alive but free from the underlying cancer and, importantly, not requiring an additional line of treatment. This seems especially important in the context of relapsed disease, since options for subsequent treatment become increasingly more limited and treatment-associated morbidity accumulates. In a recent independent study, we reported a strong correlation between PFS and OS in the first-line setting [3]. In relapsed HL, contemporary second-line treatment results in long-term survival in nearly half of the patients with 5-year PFS and OS rates of 57% and 69%, and 10-year PFS and OS rates of 48% and 60%, respectively [4]. There is, however, large heterogeneity with regards to 5-year PFS with different second-line approaches, ranging from 36% in high-risk to 73% in low-risk patients. Of note, recent data suggest an improved outcome in relapsed HL after autologous transplantation in the era of novel agents [5]. The relationship of PFS and OS in light of the relatively favorable long-term outcomes of HL in the relapsed setting, however, remains unclear. Due to the high relevance for the design of clinical trials, interpretation of trial results and approval processes of anticancer agents [6], but also for patient counseling and treatment selection, we herein aimed to evaluate the correlation of PFS and OS after treatment of relapsed HL.

Specifically, we investigated the correlation of treatment effects on PFS and OS, the correlation of risk factor (RF) effects on PFS and OS and the direct correlation of PFS and OS in HL patients in the relapsed setting. We evaluated individual patient data of 375 patients with relapsed HL treated within the prospective randomized phase III GHSG trials HDR1 (*N* = 141) and HDR2 (*N* = 234) between 02/1993 and 06/2007. Patients provided written informed consent to both trials that were performed commensurate with the Declaration of Helsinki and have previously been published [7, 8]. Both trials included patients aged 18–60 years with histologically confirmed relapsed HL after ≥1 prior treatment and naïve to high-dose chemotherapy and autologous stem-cell transplantation (ASCT). In HDR1 [7], patients received two cycles of Dexa-BEAM (dexamethasone, carmustine, etoposide, cytarabine, and melphalan) and either two further courses of Dexa-BEAM or high-dose BEAM and ASCT. In HDR2 [8], patients were assigned to two cycles of dexamethasone, cytarabine, and cisplatin (DHAP), and thereafter randomly assigned to either BEAM followed by ASCT or sequential cyclophosphamide, methotrexate, and etoposide in high doses before BEAM followed by ASCT. All patients who received at least one dose of salvage chemotherapy within the respective trial were included in the present retrospective analysis. PFS is defined as the duration between trial enrollment and the date of first progression/relapse or death for any cause. In absence of progression, relapse or death, PFS was censored at last follow-up. OS is defined as time from trial enrollment to death and censored at last follow-up. As previously described [3], we correlated treatment effects and risk factor (RF) [9] effects on PFS and OS with a marginal Cox proportional hazards regression model for multiple failure time data according to the Wei-Lin-Weissfeld (WLW) method [10], adjusted for age (dichotomized <45 and ≥45 years) and sex. Additionally, we applied copula models (Clayton, Frank, Gumbel) to estimate the correlation of PFS and OS themselves on the patient level [11–13]. Assessment of strength of correlations was based on broadly accepted ranges for the correlation measures [14]. All analysis were conducted in SAS 9.4 or later (SAS Institute, Inc.). Further details and justification of the methodology applied have been published recently in our comprehensive analysis covering the first-line setting [3].

Patient characteristics are summarized in Supplementary Table 1. With a median follow-up of 4.5 years, at least one PFS event, defined as relapse or progression of relapsed HL or death for any reason, was recorded in 146 of 375 patients (38.9%, Supplementary Table 2). At least one OS event, defined as death for any reason, was documented in 111 of 375 patients (29.6%, Supplementary Table 2). The hazard ratios for effects of treatment on PFS and OS were 0.61 (95% confidence interval, CI = 0.38–0.98) and 1.01 (95% CI = 0.63–1.63) for HDR1 and 1.1 (95% CI = 0.69–1.75) and 0.8 (95% CI = 0.44–1.47) for HDR2, respectively, as shown in Supplementary Table 3. The WLW analysis revealed strong and homogeneous correlations of impact of therapy on PFS and OS at patient level within the trials (Table 1). Pearson correlation r of the therapeutic impact on PFS and OS were r = 0.72 (95% confidence interval CI = 0.66–0.76, *P* < 0.001) in all 375 patients analyzed, r = 0.72 (95% CI = 0.63–0.79, *P* < 0.001) in the HDR1 trial and r = 0.74 (95% CI = 0.67–0.79, *P* < 0.001) in the HDR2 trial (Table 1). Correlation coefficients exceeding 0.50 are considered strong, highlighting a robust relationship between the two endpoints PFS and OS [14]. The analysis of different RFs with regards to their prognostic effects on PFS and OS (Supplementary Table 4) revealed similarly robust correlations, confirming a strong relation between PFS and OS in relapsed HL patients also in light of well-established RFs [9] (Supplementary Table 5). Based on the fit criteria of the three Archimedean copula Clayton, Frank and Gumbel, Gumbel copula demonstrated the best fit (data not shown). Correlation of PFS and OS themselves at the patient level were strong and homogeneous with Pearson r = 0.85 (95% CI = 0.82–0.88) in the HDR1 trial and r = 0.84 (95% CI = 0.81–0.86) in the HDR2 trial (Table 2).

**Table 1:** Pearson product moment correlations of treatment effects on PFS and OS.

Trial	*N*	Pearson r	LL 95%CI	UL 95%CI	*P*
HDR1	141	0.72	0.63	0.79	<0.001
HDR2	234	0.74	0.67	0.79	<0.001
Total	375	0.72	0.66	0.76	<0.001

*PFS* progression-free survival, *OS* overall survival, *N* number of patients, *LL* lower limit of 95% confidence interval, *UL* upper limit of 95% CI.

**Table 2:** Correlation measures of PFS and OS themselves at the patient level with Gumbel copula.

Trial	Kendall’s tau	LL 95%CI	UL 95%CI	Pearson r	LL 95%CI	UL 95%CI	Spearman rho	LL 95%CI	UL 95%CI
HDR1	0.65	0.61	0.68	0.85	0.82	0.88	0.84	0.81	0.87
HDR2	0.63	0.61	0.66	0.84	0.81	0.86	0.83	0.80	0.85

*PFS* progression-free survival, *OS* overall survival, *LL 95%CI* lower limit of 95% confidence interval, *UL* upper limit of 95% confidence interval.

The present study of 375 patients with relapsed HL treated within two randomized trials revealed strong correlations between treatment and RF effects on PFS and OS. Moreover, the survival measures PFS and OS showed a strong correlation at the individual patient level. These observations including the magnitude of the correlation measures are very well in line with our recent report in the first-line HL treatment setting [3]. In HL, strategies to reproducibly measure minimum residual disease (MRD) either by circulating tumor DNA or soluble biomarkers such as TARC are actively explored. To this end, current data is, however, mostly derived in the first-line setting. It is hence still unclear whether e.g. MRD-negativity or other non-survival endpoints could potentially serve as surrogates for OS in relapsed HL in the future. This underscores the importance of PFS as a well-established, broadly accepted and robustly measurable primary endpoint for clinical trials and also for the counseling of patients.

To apply widely recommended and acknowledged methods to investigate correlations of PFS and OS [15], we limited the present analysis to data from randomized phase III clinical trials, which are rare in an orphan disease such as HL. While this ensures a robust analysis, the following potential limitations arise: The available trials were conducted before the approval of targeted agents such as brentuximab vedotin or the anti-PD1 antibodies nivolumab and pembrolizumab for relapsed HL. Additionally, they only included patients up to the age of 60 years. Generalizability of our results to the contemporary treatment setting and also the real-world scenario hence needs to be determined ideally by future analyses of data from more contemporary randomized trials. In light of the heterogeneous outcomes after second-line treatment in the real-world setting [4] and a rapidly changing treatment landscape, planning of future trials including power calculations to show (or exclude) survival differences remains challenging. Our results, however, support the use of PFS as primary endpoint for future trials e.g. testing whether ASCT can be omitted in a subset of patients. A potential further limitation is the assumption of proportional hazards, which is the basic requirement of Cox proportional hazards models. Importantly, in our study the robust variance estimates by WLW accounted for possible deviations and tests of the proportional hazards assumption did not show non-random deviations.

In summary, PFS and OS as well as treatment and RF effects on PFS and OS are strongly correlated in patients treated for relapsed HL. Confirming recent results in the setting of HL first-line treatment, PFS thereby constitutes a highly relevant endpoint also in the setting of relapsed HL.

## Supplementary information

Supplemental Material
