# Correction: Correlation of progression-free survival and overall survival after treatment for relapsed Hodgkin lymphoma: individual patient data analysis of randomized German Hodgkin Study Group (GHSG) Trials

**DOI:** 10.1038/s41375-025-02597-4

**Published:** 2025-03-30

**Authors:** P. J. Bröckelmann, H. Müller, M. Fuchs, S. Gillessen, D. A. Eichenauer, S. Borchmann, A. S. Robertz, K. Behringer, J. Welters, J. Ferdinandus, B. Böll, H. Tharmaseelan, X. Yang, C. Kobe, H. T. Eich, C. Baues, W. Klapper, P. Borchmann, B. von Tresckow

**Affiliations:** 1 University of Cologne, Faculty of Medicine and University Hospital of Cologne, Department I of Internal Medicine, Center for Integrated Oncology Aachen Bonn Cologne Düsseldorf (CIO ABCD) and German Hodgkin Study Group (GHSG), Cologne, Germany; 2 Merck & Co., Inc, Kenilworth, NJ USA; 3 Department of Nuclear Medicine, University Hospital of Cologne, Cologne, Germany; 4 Department of Radiation Oncology, University Hospital of Muenster, Muenster, Germany; 5 Department of Radiotherapy, University of Bochum, Bochum, Germany; 6 Hematopathology Section and Lymph Node Registry, Department of Pathology, University Hospital Schleswig-Holstein, Campus Kiel, Kiel, Germany; 7 Department of Hematology and Stem Cell Transplantation, West German Cancer Center and German Cancer Consortium (DKTK Partner Site Essen), University Hospital Essen, University of Duisburg-Essen, Essen, Germany

**Keywords:** Hodgkin lymphoma, Clinical trials

Correction to: *Leukemia* 10.1038/s41375-025-02567-w, published online 24 March 2025

In this article the author’s name D. A. Eichenauer. was incorrectly written as D. Eichenauer. The original article has been corrected.

