# Supplementary material for: Correlation of progression-free survival and overall survival after treatment for relapsed Hodgkin lymphoma: individual patient data analysis of randomized German Hodgkin Study Group (GHSG) Trials
Source: Leukemia. 2025 Mar 24;39(4):988–90. doi: 10.1038/s41375-025-02567-w (PMC11976262; doi:10.1038/s41375-025-02567-w)
Supplement: Supplementary file 1 — Supplemental Material [file 41375_2025_2567_MOESM1_ESM.docx]

**Supplementary Material**

**Supplementary Tables**

**Suppl. Table 1: Patient Characteristics**

|  | HDR1 trial (N=141) | HDR2 trial (N=234) | Total (N=375) |
| --- | --- | --- | --- |
| Sex |  |  |  |
| female | 52 (37%) | 82 (35%) | 134 (36%) |
| male | 89 (63%) | 152 (65%) | 241 (64%) |
| Age ≥ 45 years | | | |
| no | 120 (85%) | 190 (81%) | 310 (83%) |
| yes | 21 (15%) | 44 (19%) | 65 (17%) |
| Treatment |  |  |  |
| A | 65 (46%) | 117 (50%) | 182 (49%) |
| B | 76 (54%) | 117 (50%) | 193 (51%) |
| Ann Arbor stage IV | | | |
| no | 81 (57%) | 174 (74%) | 255 (68%) |
| yes | 60 (43%) | 60 (26%) | 120 (32%) |
| Bulk tumor ≥ 5cm | | | |
| Missing | 9 | 0 | 9 |
| no | 106 (80%) | 162 (69%) | 268 (73%) |
| yes | 26 (20%) | 72 (31%) | 98 (27%) |
| ECOG ≥1 |  |  |  |
| Missing | 23 | 2 | 25 |
| no | 98 (83%) | 154 (66%) | 252 (72%) |
| yes | 20 (17%) | 78 (34%) | 98 (28%) |
| Anemia^#^ |  |  |  |
| Missing | 20 | 1 | 21 |
| no | 90 (74%) | 195 (84%) | 285 (81%) |
| yes | 31 (26%) | 38 (16%) | 69 (19%) |
| Inadequate response to salvage chemotherapy^$^ | | | |
| Missing | 15 | 0 | 15 |
| no | 42 (33%) | 61 (26%) | 103 (29%) |
| yes | 84 (67%) | 173 (74%) | 257 (71%) |
| ^#^hemoglobin<10.5 g/dl in females and <12.0 g/dl in males; ^$^no complete remission; | | | |

**Suppl. Table 2: PFS and OS events**

|  |  | Progression free survival (PFS) | | | Overall survival (OS) | | |
| --- | --- | --- | --- | --- | --- | --- | --- |
| Trial | N | events | censored | % censored | events | censored | % censored |
| HDR1 | 141 | 74 (52%) | 67 | 47.5 | 68 (48%) | 73 | 51.8 |
| HDR2 | 234 | 72 (31%) | 162 | 69.2 | 43 (18%) | 191 | 81.6 |
| Total | 375 | 146 (39%) | 229 | 61.1 | 111 (30%) | 266 | 70.4 |

PFS= progression-free survival, OS= overall survival

**Suppl. Table 3: Treatment effects on PFS and OS**

|  |  | Progression free survival (PFS) | | | | Overall survival (OS) | | |
| --- | --- | --- | --- | --- | --- | --- | --- | --- |
| Trial | N | HR | LL  95%CI | UL  95%CI | | HR | LL  95%CI | UL 95%CI |
| HDR1 | 141 | 0.61 | 0.38 | 0.98 | 1.01 | | 0.63 | 1.63 |
| HDR2 | 234 | 1.10 | 0.69 | 1.75 | 0.80 | | 0.44 | 1.47 |

PFS= progression-free survival, OS= overall survival, N= number of patients, LL = lower limit of 95% confidence interval, UL= upper limit of 95% CI

**Suppl. Table 4: Prognostic effect of established RF on PFS and OS**

|  |  |  |  | | | PFS | |  |  | | | OS | |  |
| --- | --- | --- | --- | --- | --- | --- | --- | --- | --- | --- | --- | --- | --- | --- |
|  | Risk factor | N | | HR | LL 95%CI | | UL 95%CI | | | HR | LL 95%CI | | UL 95%CI | |
|  | Ann Arbor Stage IV | 375 | | 2.05 | 1.48 | | 2.83 | | | 2.39 | 1.65 | | 3.48 | |
|  | ECOG ≥ 1 | 350 | | 1.59 | 1.09 | | 2.30 | | | 1.45 | 0.92 | | 2.29 | |
|  | Bulk ≥ 5 cm | 366 | | 1.15 | 0.79 | | 1.67 | | | 1.40 | 0.91 | | 2.15 | |
|  | Anemia^#^ | 354 | | 2.01 | 1.37 | | 2.93 | | | 2.03 | 1.34 | | 3.08 | |
|  | Inadequate response to salvage chemotherapy^$^ | 360 | | 1.51 | 1.02 | | 2.23 | | | 1.82 | 1.11 | | 2.97 | |
|  | ^#^hemoglobin<10.5 g/dl in females and <12.0 g/dl in males; ^$^no complete remission; PFS= progression-free survival, OS= overall survival, N= number of patients, HR= hazard ratio, LL = lower limit of 95% confidence interval, UL= upper limit of 95% CI | | | | | | | | | | | | | |

**Suppl. Table 5. Pearson product moment correlations of prognostic effects of risk factors on PFS and OS**

| Risk factor | N | Pearson r | LL 95%CI | UL 95%CI |
| --- | --- | --- | --- | --- |
| Ann Arbor Stage IV | 375 | 0.72 | 0.67 | 0.77 |
| ECOG ≥ 1 | 350 | 0.74 | 0.68 | 0.78 |
| Bulk ≥ 5 cm | 366 | 0.72 | 0.66 | 0.76 |
| Anemia# | 354 | 0.73 | 0.68 | 0.77 |
| Inadequate response to  salvage chemotherapy$ | 360 | 0.73 | 0.68 | 0.77 |

#hemoglobin<10.5 g/dl in females and <12.0 g/dl in males; $no complete remission; PFS= progression-free survival, OS= overall survival, N= patient number, LL = lower limit of 95% confidence interval, UL= upper limit of 95% CI
